# Utilizing a new self-centering hysteresis model to assess the seismic vulnerability of a long-span cable-stayed bridge equipped with SMA wire-based roller bearings

**DOI:** 10.1186/s43251-022-00064-z

**Published:** 2022-10-02

**Authors:** Shuai Li, Hedayati Dezfuli Farshad, Jing Quan Wang, M. Shahria Alam

**Affiliations:** 1 School of Civil Engineering, Southeast University, Nanjing, Jiangsu, 210096 China; 2 Bridge Engineer and Analyst; Parsons, Burnaby, BC V5H 4M2 Canada; 3 School of Engineering, The University of British Columbia, Kelowna, BC V1V1V7 Canada

**Keywords:** Shape memory alloy, SMA wire-based roller bearing, Seismic vulnerability analysis, Cable-stayed bridge, Near fault ground motion

## Abstract

A novel shape memory alloy wires-based smart roller bearing (SMA-RBs) has been developed and its cyclic behavior under reverse cyclic loadings has been experimentally investigated. However, its efficacy and performance in enhancing the seismic performance of bridge structures have not been well understood and proven. A new self-centering hysteresis model for SMA-RBs has been proposed to properly simulate their hysteretic behavior, which has been experimentally validated through a pseudo-static test. A methodology is proposed to determine the four damage states of SMA-RB (i.e. slight, moderate, extensive, and collapse) considering the contribution of SMA wires. The smart SMA-RBs are utilized for a cable-stayed bridge in China. The vulnerability of two reference bridges, i.e. the floating system (FS) and rigid system (RS), and one isolated bridge equipped with SMA-RBs (SMA-RBS) are compared at component and system levels. The applicability of three commonly used intensity measures (IMs), i.e. PGA, PGV, and Sa(T_1_), are evaluated and PGV turns out to be the optimal IM for long-span cable-stayed bridge systems. Results show that incorporating SMA wires in roller bearings can decrease the failure probabilities of the bearing. The piers and towers with SMA-RBs lead to lower seismic fragility over the towers and piers in the reference bridges. The RS is the most vulnerable bridge whereas the SMA-RBS is the least vulnerable bridge among the four bridges. The SMA-RBS experience a much lower collapse damage probability compared to RS ad FS.

## Introduction

In the last few decades, seismic isolation systems have been extensively implemented to reduce the seismic damage of bridge structures located in high-seismic regions (Zheng et al. ; Hedayati Dezfuli et al. 2017). However, the conventional isolation devices have several disadvantages such as long-term performance (durability, aging, and maintenance), instability due to large deformation, and inadequate self-centering capacity (Hedayati Dezfuli and Alam 2016; Zheng et al. 2018). To partially address these limitations, shape memory alloy (SMA)-based isolation bearings have been developed by several researchers (Choi et al., 2005; Attanasi et al., 2009; Ozbulut & Hurlebaus, 2011; Hedayati Dezfuli & Alam, 2013; Mishra et al., 2015; Zheng et al. 2018; Narjabadifam et al. 2020). Compared to other metallic materials, SMAs possess high strength, good fatigue and corrosion resistance, superelasticity, and energy dissipation (Attanasi et al., 2009; Qiu and Zhu 2017; Wang et al. 2020; Ozbulut & Hurlebaus, 2011). Therefore, SMAs can improve the self-centering properties and energy dissipation capability of conventional isolators. In these smart isolation bearings, SMAs are installed/wrapped around the conventional bearings in the forms of bars (Alam et al. 2012; Bhuiyan and Alam 2012; Desroches and Delemont 2002; Wilde et al. 2000), wires (Attanasi et al. 2009; Bhuiyan and Alam 2013; Hedayati Dezfuli and Alam 2016; Mishra et al. 2015; Ozbulut and Hurlebaus 2010; Xue and Li, 2007), cables (Zheng et al., 2018, 2019; Cao et al. 2020; Li et al. 2020a, b), and springs (Attanasi et al., 2009). It is of great interest to use such novel bearings in mitigating seismic damage to bridge structures.

In these novel SMA wire-based isolation bearings, SMA wires or cables were incorporated into various isolators with various arrangements, including the straight, cross, and double-cross configurations (Hedayati Dezfuli and Alam 2016; Liang et al. 2020; Cao and Yi 2021; Pang et al. 2021; Cao et al. 2022; Fang et al. 2022; Zheng et al., 2021, 2022). In most of the previous studies, the combination of the bilinear model for conventional isolators and the flag-shaped model for SMA was generally used to simulate the hysteretic behavior of such smart bearings (Hedayati Dezfuli and Alam, 2016, 2017; Zheng et al. 2018; Pang et al. 2021). However, the experimental studies by the authors have proved that the self-centering characteristics of such smart bearings cannot be correctly reflected by using the flag-shaped model without considering the effect of wire configurations (Li et al. 2022). In their latest studies, a triangular-shaped constitutive model has been proposed to accurately describe the behavior of SMA-based bearings considering different wire configurations (Hedayati Dezfuli and Alam, 2015; Hedayati Dezfuli et al. 2017; Li et al. 2022).

The SMA-based bearings were mainly implemented in short or medium-span highway bridges (Wilde et al. 2000; Ozbulut and Hurlebaus 2010; Hedayati Dezfuli and Alam 2016). A series of experimental and numerical investigations have shown that SMA-based bearings can efficiently enhance the seismic performance of short or medium-span highway bridges. However, the feasibility of such smart bearings in reducing the seismic damage of long-span bridges has not been thoroughly studied. Successful implementation of SMA-based bearings in long-span bridges requires a complete performance-based evaluation of this structural system in the light of performance-based earthquake engineering. To the best of the authors’ knowledge, no study has been directed towards understanding the performance of the long-span bridge equipped with SMA-based bearings considering a new self-centering hysteretic model.

The high cost of SMA materials hinders the wide application of SMA-based bearing. To tackle this challenge, a new type of smart isolator with a high benefit-to-cost ratio, i.e., SMA wire-based roller bearing (SMA-RB), was developed by the authors (Li et al. 2022). As the cost of the rollers is much lower than that of the conventional viscous dampers, lead rubber bearing, and friction-based isolators, it is possible to have a final cost that is in the order of that of conventional isolators. Compared to other SMA-based isolation bearings (such as rubber-based dampers and friction bearings), the smart roller bearings possess the following advantages. (1) The initial cost of the rollers is much lower than the rubber bearing or the sliding friction bearing. (2) It is convenient to maintain and replace the rollers in the smart bearing. (3) Compared to the rubber bearing, the rollers using weathering-resistant steel have higher durability against the harsh environment. The cyclic behavior of SMA-RBs has been theoretically and experimentally investigated.

This study aims at assessing the efficacy of SMA-RBs in mitigating the seismic damage of long-span bridges. A newly proposed constitutive model for SMA-RBs is used to describe their hysteretic behavior. The new constitutive model is coded in OpenSees software (McKenna et al., 2000). A cable-stayed bridge located in China is taken as an example considering different types of deck-tower connections. Conventional roller bearing and smart roller bearing, i.e. SMA-RB are chosen as the isolation bearings. 20 near-fault ground motions with a wide range of PGA in two orthogonal directions are chosen in the numerical simulations. The limit states of SMA-RB are defined according to the limit states of roller bearing as well as the strain of SMA wires. The fragility curves of the pier, cable, tower, and bearing are generated using the newly self-centering hysteretic model. Finally, the system-level fragility curves are calculated by combining the effects of these four major components based on a serial and a parallel system, respectively. The upper bounds in the case of the serial and parallel systems are used to conservatively estimate the fragility of the bridge system.

## Smart seismic isolation system

**Fig. 1:**
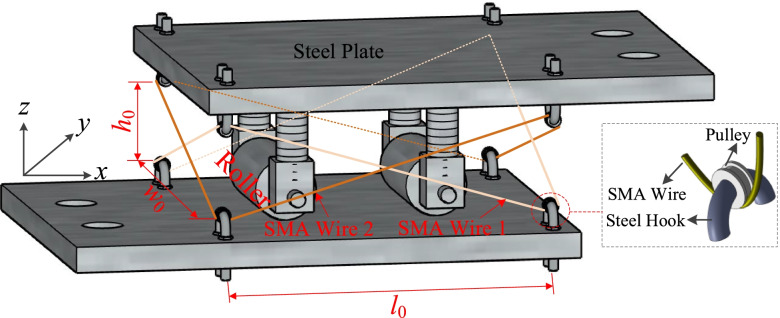
SMA-RB system in a cross configuration

**Fig. 2:**
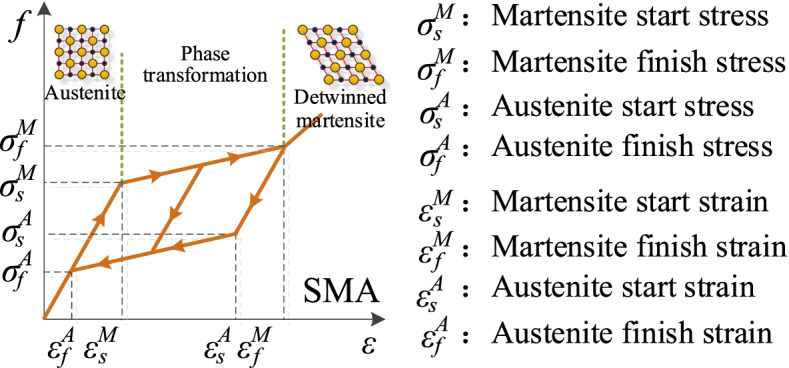
the constitutive model of SMA (Auricchio 2001)

**Fig. 3:**
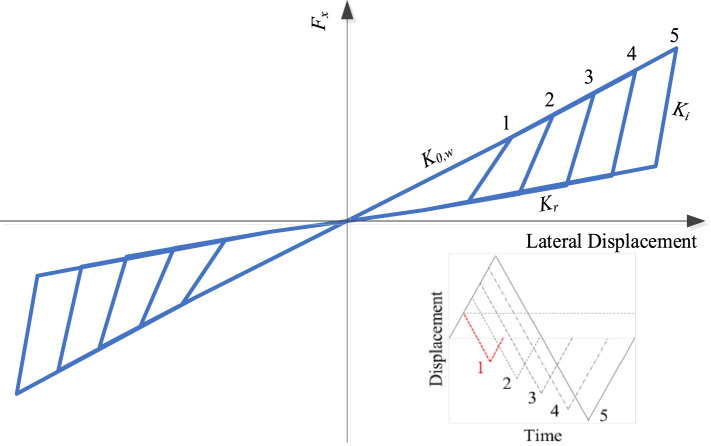
The constitutive model of C-SMAW (Hedayati Dezfuli and Alam 2015 and Li et al. 2022)

A smart roller bearing (RB) equipped with cross SMA wires is used in this research. This smart bearing contains two parts: RB and cross SMA wires (see Fig. 1). To prevent SMA wires at the connection of steel hooks from experiencing plastic deformation due to stress concentration, a pulley within the steel hook, which can turn around the steel hook freely, is designed and used in this smart bearing. A groove with a certain depth is notched on the surface of the pulley (see Fig. 1), which can ensure a reliable connection between the pulley and SMA wires. A superposition method is considered to develop the constitutive model of SMA-RB (Fig. 2). The hysteretic model of RBs is described using a bilinear model. The hysteretic model of C-SMAW can be developed by calculating the force and deformation in wires according to the special wire configuration (cross). Here, the nonlinear behavior of SMAs is described using the flag-shaped hysteretic model (Fig. 2). A detailed working mechanism can be found in the study by Li et al. (2022). The hysteretic response of C-SMAW can be obtained and plotted in Fig. 3. It can be seen that the model can be characterized by initial stiffness (*K*_0,*w*_), intermediate stiffness (*K*_*i*_), and re-centering stiffness (*K*_*r*_). The accuracy of the proposed hysteresis model has been experimentally validated (Li et al. 2022).

**Fig. 4:**
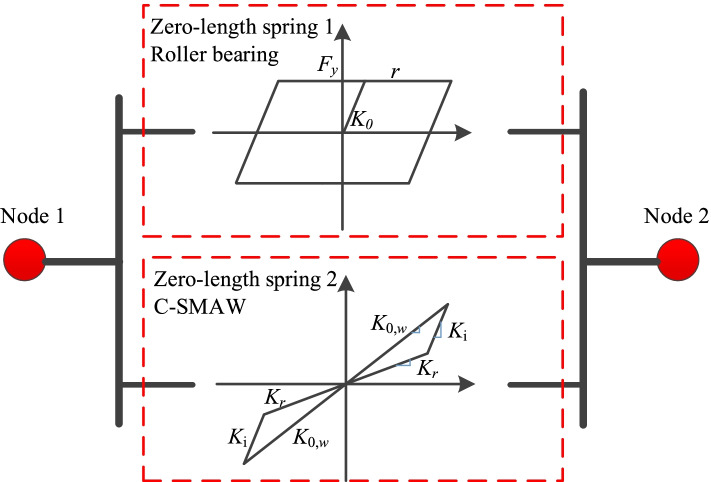
Numerical model of SMA-RB system

The constitutive model of SMA-RB can be obtained by combining the bilinear model for RB with the self-centering model for C-SMAW, as shown in Fig. 3. new User Elements for C-SMAW in OpenSees have been generated using the new proposed model. Two zero-length elements are combined in parallel to simulate the SMA-RB (see Fig. 4).

## Case study: bridge description and numerical models

**Fig. 5:**
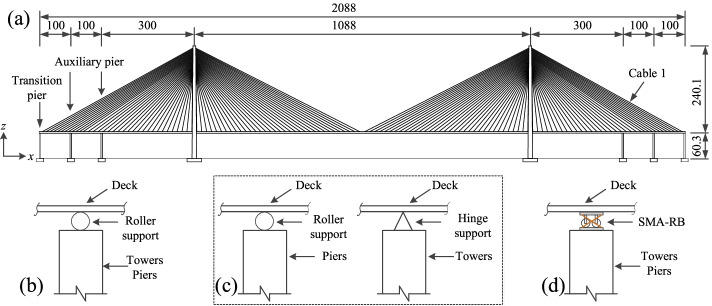
The detailed geometry of the cable-stayed bridge (meters) (**a**) Bridge model, (**b**) Floating system (FS), (**c**) Rigid system (RS), and (**d**) SMA-RB system (SMA-RBS)

A typical cable-stayed bridge located in China (see Fig. 5) is used to develop fragility functions and assess the efficacy of SMA-RBs based on the new constitutive model. Two different types of bridges are taken into account in the study. Two common types of floating system (FS) and rigid system (RS) are defined as reference bridges. For the bridge equipped with SMA-RBs, the novel smart bearings are installed at pier and tower locations. Since the SMA-RBs have superior energy dissipation capacity, they can be regarded as dampers. Besides, many researchers have proposed that the over-displacement of the deck should be addressed to ensure the seismic safety of a cable-stayed bridge under strong earthquakes (Ye et al. 2004). Hence, in this study, the SMA-RBs are regarded as both dampers and restrainers.

OpenSees is utilized to generate the three-dimensional (3D) numerical model of the bridge. The towers and piers are modeled using displacement-based nonlinear fiber elements. The properties of the unconfined concrete, confined concrete, and reinforcing steel are defined, respectively. C50 and C40 concrete are considered for the tower and pier, respectively. HRB335 and HRB400 are used as the stirrup and longitudinal rebar, respectively. Concrete01 is used to model the concrete fibers and ReinforcingSteel material is chosen to stimulate the reinforcing steel fibers. The constitutive behavior of the longitudinal reinforcement is modeled using a uniaxial material hysteretic model (Chang and Mander 1994). The yielding strength, ultimate stress, and elastic modulus of the longitudinal rebars are 400 MPa, 527 MPa, and 200 GPa, respectively. The corresponding yielding and ultimate strains are 0.0017 and 0.09, respectively. The Kent-Scott-Park model (Kent and Park 1971) defined in Concrete01 material is used to describe the stress–strain relationship of concrete in compression. The compressive strengths of the concrete for the tower and pier are 50 MPa and 40 MPa, respectively. The corresponding strain at peak stress is 0.2%. The girder is discretized based on the suspended points of the stayed cables. The steel girders are simulated by the elastic beam-column elements. The mass of each segment is assumed to be equally distributed between two adjacent nodes in the form of point mass. 3D tension-only truss elements considering the influence of sag are used to model the cables. The nonlinear behaviors of the cable stays are idealized using the Ernst equation of equivalent modulus of elasticity. The cables are prestressed and the property of each cable at different locations has been listed in the study by Li et al. (2017). More detailed material properties and boundary conditions of the bridge are available in the studies by Li et al. (2016 and 2017). In the FE models, it is assumed that the towers and piers are fixed to their foundations since the bridge is supported by bedrock.

SMA-RBs are designed and installed at the locations of the pier and tower. Following the studies by Hedayati Dezfuli and Alam (2015) and Li et al. (2022), the SMA wires wrapped around the conventional isolators can provide enough resultant forces to prevent the uplift of the bearing under earthquakes. As a result, SMA-RBs possess superior energy dissipation capacity when implemented in a cable-stayed bridge. The design methodology for roller bearing and SMA-RB can be found in Li et al. (2022). A new type of SMA, i.e. the iron-based SMA (FeNiCoAlTaB) wires, is implemented in the SMA-RB. This is because the iron-based alloy has a higher energy dissipation capacity and lower austenite finish temperature (-62 °C) compared to nickel-titanium (NiTi) alloy. The SMA wires can remain superelastic within a wide range of temperatures (Hedayati Dezfuli and Alam 2017). A total of 54 SMA-RBs are arranged in the bridge. 3 bearings are installed at the top of each pier and 9 bearings are arranged between the girder and tower. Zero-length elements are used to model the bearings in which the SMA model and the LRB model are acting in parallel. The mechanical properties of RB are idealized as a bilinear model. The initial elastic stiffness and frictional coefficient of the roller bearing are 14.8 kN/mm and 0.2, respectively. The values of *K*_0,*w*_, *K*_*i*_, and *K*_*r*_ of C-SMAW are 4.66 kN/mm, 16.42 kN/mm, and 2.31 kN/mm, respectively.

The fundamental natural periods of the floating system (FS), rigid system (RS), and the bridge equipped with SMA-RBs (SMA-RBS) are 16.27, 4.43, and 9.8 s, respectively. The first modes of the FS and SMA-RBS are the longitudinal floating vibration. The first mode of the RS is the vertical bending vibration. Compared to FS and SMA-RBS, the RS has lower periods because of the higher lateral stiffness of the hinge support at the tower location and as a result, the flexibility of the bridge is reduced.

## Seismic vulnerability analysis

Seismic fragility defines the damage probability of a structure at a given intensity measure (IM), i.e. the probability levels that the structural seismic demand exceeds its capacity. The fragility functions can be derived by using the Probabilistic Seismic Demand Model (PSDM). The relationship between the engineering demand parameters (EDP) and the intensity measures (IM) of the ground motions are shown in Eq. 1.

1 $$EDP={a\left(IM\right)}^{b}$$

where, *a* and *b* are regression coefficients. This study models the fragility of each vulnerable component as a standard normal cumulative distribution function at each damage state.

2 $$P\left[LD|IM\right]=\Phi \left[\frac{\mathrm{ln}\left(EDP/{S}_{c}\right)}{\sqrt{{\beta }_{D|IM}^{2}+{\beta }_{c}^{2}}}\right]$$

where, *S*_c_ and *β*_c_ are the median estimate and standard deviations of the structural capacity, respectively. The standard deviation of the demand, *β*_D|IM_, is calculated as follows:

3 $${\beta }_{D|IM}=\sqrt{\frac{{\sum }_{i=1}^{N}{\left[\mathrm{ln}\left({EDP}_{i}\right)-\mathrm{ln}\left({aIM}_{i}^{b}\right)\right]}^{2}}{N-2}}$$

where, *N* is the total number of simulation cases.

For a cable-stayed bridge system, towers, piers, bearings, and cables can experience different damage states and as a result, it is difficult to evaluate the global damage states based on only one component. Here, the system fragility is calculated by assuming that the bridge operates like a serial or a parallel system. The system fragility should be located in-between the fragilities of the serial and parallel system. According to the previous studies, the system fragilities, *P*(*F*_system_) can be determined as Eq. 6 (Zhang and Huo 2009; Hedayati Dezfuli and Alam 2016). Here, the bridge failure probability can be conservatively assessed by using the maximum values of the upper bounds in the two systems.

4 $$\prod_{i=1}^{n}\left[P\left({F}_{i}\right)\right]\le P\left({F}_{system}\right)\le 1-\prod_{i=1}^{n}\left[1-P\left({F}_{i}\right)\right]$$

**Fig. 6:**
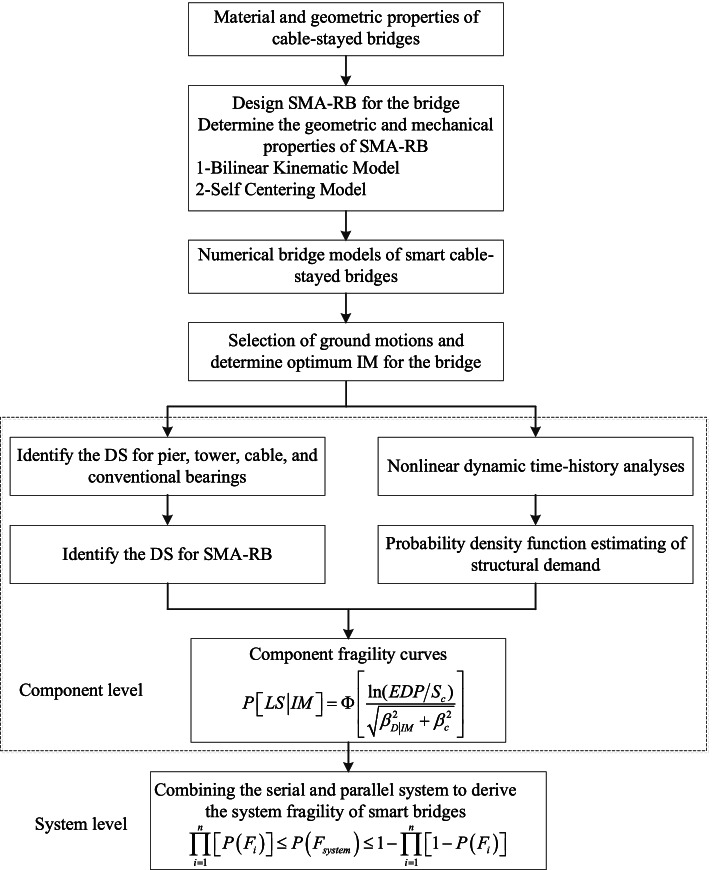
Flowchart of the proposed methodology for smart cable-stayed bridges

A flowchart for deriving the fragility curves of the bridge equipped with SMA-RBs is proposed at the component and system levels (see Fig. 6). The newly developed constitutive model of SMA-RB is used to establish the fragility functions. The identification of damage states for SMA-RB will be provided in the following section.

### Damage states

#### Damage states of the tower, pier, cable, and conventional bearing

Here, the damage states of each bridge component are described by slight, moderate, extensive, and collapse (Federal Emergency Management Agency (FEMA), 2003). In this study, four main vulnerable components are considered, including the cables, piers, towers, and bearings. The over-displacement of the girder may cause the pounding problem. Controlling the deck displacement is important to ensure the seismic safety of the bridge. However, since the pounding between the deck and the approach road of the bridge is not considered, the displacement of the deck is not selected as the indices in this study. Besides, it is expected that the deck will remain elastic under earthquakes. Hence, the deck is not regarded as the vulnerable component in the fragility analysis of a cable-stayed bridge according to previous studies (Zhong et al. 2017; Wen et al. 2021).

**Fig. 7:**
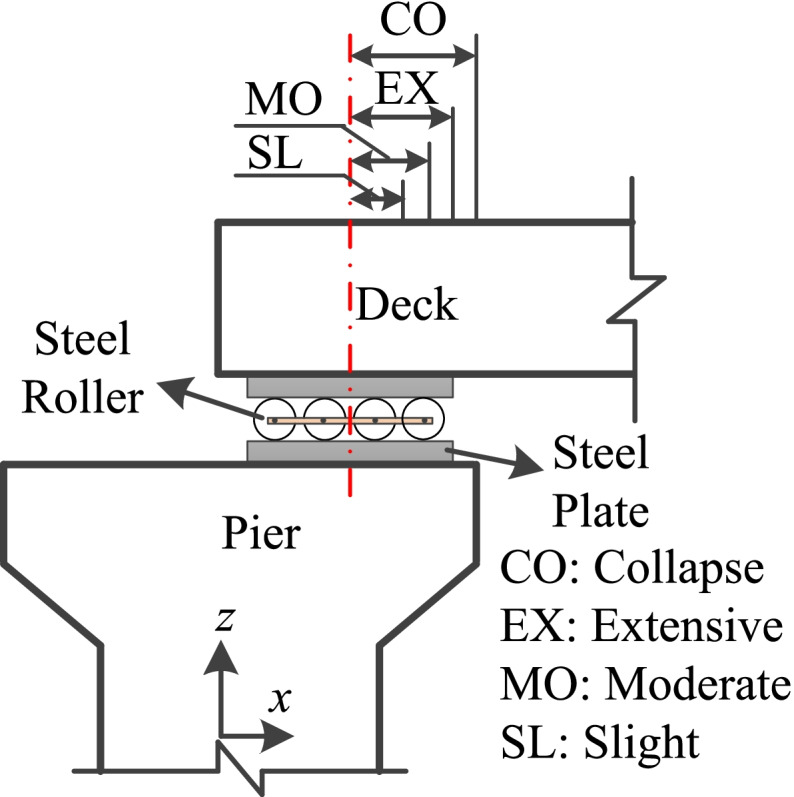
Damage states of roller supports

The tower is one of the main vulnerable components in a cable-stayed bridge. Generally, the dimensionless indices (e.g., the drift ratio) are selected to identify the damage states of the tower (Pang et al. 2013; Zhong et al. 2017; Wen et al. 2021). The force-based damage states cannot accurately reflect the capacity of the tower when it goes into the ductile range. In this regard, the drift ratio, *θ*, at the base of the tower used is chosen as the tower’s damage index. The relative displacement is defined as the damage index of roller bearing (see Fig. 7). The limit value of slight damage equals the maximum allowable displacement of the bearing under normal service conditions. The distance between the bearing’s center and the end of the steel plate and half of the distance are defined as the critical values of extensive and moderate damage, respectively. When the bearing’s center exceeds the end of the pier cap, it is assumed that the roller bearing reaches collapse damage. Displacement ductility, *μ*_d_, is considered the damage index of the bridge piers. The limit states proposed by Hwang et al. (2001) are utilized to describe their damage levels. Zhong et al. (2017) used the cable strain ductility (*ε*/*ε*_y_), which is defined as the ratio of the maximum strain to yield strain of the steel cable, to develop cable fragility curves. Since the cables have been pre-tensioned to about 32% of their yield strain in SCB, their limit states are assumed as 0.45, 0.60, 0.75, and 0.9 at different damage levels in this study. The existing models and corresponding limit states used in previous studies are shown in Table 1.

**Table 1:** Damage states of each vulnerable component

Bridge Component	EDP	Limit States				Reference
		Slight	Moderate	Extensive	Collapse	
Cable	*ε*/*ε*_y_	0.45	0.60	0.75	0.90	Zhong et al. 2017
Pier	*μ*_d_	1.00	1.20	1.76	4.76	Hwang et al., 2001
Tower	*θ*	0.007	0.015	0.025	0.050	Pang et al. 2013
Roller bearing	Δ (mm)	120	180	240	300	Assumed

#### Identification of the damage states for SMA-RB

The damage states of SMA-RB are defined considering the contributions of SMA and roller bearing in this section. The damage states of SMA-RB should be determined based on the limit states of roller bearing as well as the strain of SMA wires, *ε*_*SMA*_, at each damage state of roller bearing in Table 1. Here, a procedure is suggested to identify the damage states of SMA-RB.

**Step 1:** Calculate the strain of SMA wires, *ε*_*SMA,DS*_, at each damage state of the roller bearing (*γ*_*RB,DS*_) according to the material and geometry of the roller bearing and C-SMA wires.

**Step 2:** Check the functionality of SMA-RB.

Here, it is considered that if one of the components in SMA-RB fails, the smart isolation bearing will lose its functionality. The strain of C-SMA wires at each damage state is compared with the superelastic strain, *ε*_*s*_, to determine the functionality of SMA-RB. Two cases are considered as follows.

Case I:

5 $${\varepsilon }_{{SMA,DS}_{4}}<{\varepsilon }_{s}$$

Case II:

6 $${\varepsilon }_{{SMA,DS}_{i-1}}<{\varepsilon }_{s}<{\varepsilon }_{{SMA,DS}_{i}} \left(i=1, 2, 3, 4\right)$$

where, *DS*_*i*_ is the *i*th damage state of roller bearing; *ε*_*SMA,DSi*_ is the strain of C-SMA wires at *i*th damage state of roller bearing.

If the strain of SMA wires at collapse damage state of the roller bearing is smaller than the superelastic strain of SMA wires (Case I in Eq. 5), SMA wires will remain superelastic before the roller bearing collapses. If not (Case II in Eq. 6), the failure of SMA wires will be earlier than the collapse of roller bearing and consequently, the collapse damage of SMA-RB will be determined by SMA wires.

**Step 3:** Determine the damage states of SMA-RB.

In case I, SMA-RB has the same limit states as the roller bearing.

Case I:

7 $${DS}_{SMA-RB}={DS}_{RB}$$

In case II, the collapse damage state of SMA-RB equals the maximum displacement of roller bearing, ${DS}_{{LRB,\varepsilon }_{s}}$, when C-SMA wire reaches superelastic strain.

Case II:

8-1 $${DS}_{SMA-RB}={DS}_{RB}\left({\varepsilon }_{SMA}<{\varepsilon }_{s}\right)$$

Collapse:

8-2 $${DS}_{SMA-RB}={DS}_{{RB,\varepsilon }_{s}}\left({\varepsilon }_{SMA}={\varepsilon }_{s}\right)$$

**Fig. 8:**
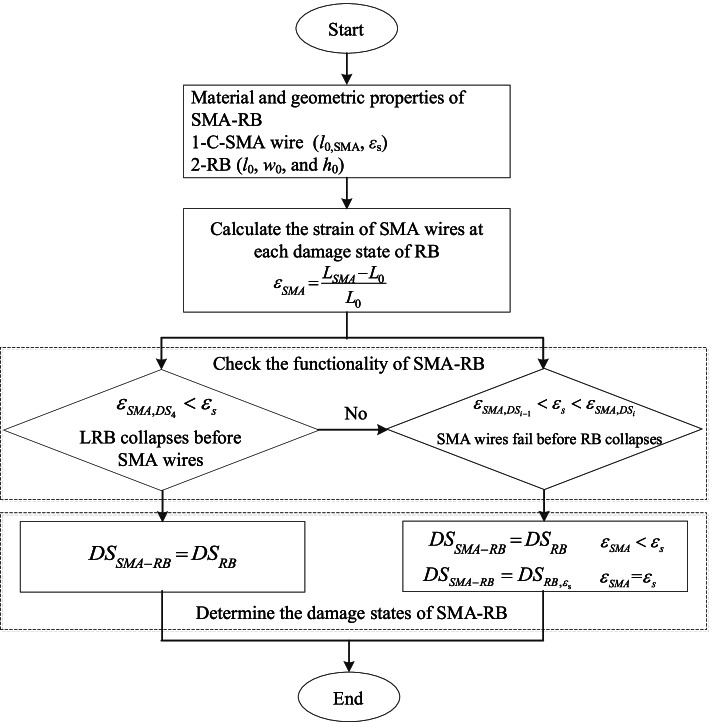
Flowchart for determining the damage states of SMA-RB

For instance, in case II, when *i* = 4, i.e. *ε*_*SMA,DS3* <_ *ε*_*s* <_ *ε*_*SMA,DS4*_, SMA wires exceeding superelastic strain range will experience permanent strain and cannot fully recover at collapse damage state of roller bearing. Hence, the damage states of SMA-RB are the same as those of roller bearing at each limit state. The shear strain of SMA-RB at collapse damage should be determined by that of SMA-RB at the superelastic strain of SMA wires. A flowchart for determining the damage states of SMA-RB is illustrated in Fig. 8.

According to the proposed procedure, the maximum strain of SMA wires used in this study is smaller than the superelastic strain of FeNiCoAlTaB SMA wires (around 13.5%). SMA wires are functional before the roller bearing collapses. Hence, the four limit states of SMA-RB are the same as those of roller bearing in this study (see Table 1).

### Near fault ground motions and selection of intensity measures

Here, a set of 20 near-fault earthquake records (having two horizontal components for each record) are selected as the input. The fault distances of these records range from 0.5 to 12.4 km. The magnitudes of these records are between 6.0 and 8.0. Table 2 lists the basic properties of each ground motion. In this study, the scaling approach is implemented for developing the fragility functions. Scaling factors ranging from 0.5 to 5.0 with the step of 0.5 are selected to cover a wide range of damage levels and increase the accuracy of the results for developing fragility curves. An adequate number of data (about 200) are generated to establish the fragility curves of the considered bridge.

**Table 2:** Basic properties of the near-fault ground motions

No	Earthquake	Year	Magnitude (Richter)	*R*_rup_ (km)	PGA (g)		PGV (cm/s)	
					*x*	*y*	*x*	*y*
1	Chi-Chi, Taiwan	1999	7.6	1.8	0.35	0.42	159.0	118.5
2	Chi-Chi, Taiwan	1999	7.6	2.5	0.81	0.6	126.2	78.8
3	Chi-Chi, Taiwan	1999	7.6	3.0	0.51	0.37	279.9	264.0
4	Chi-Chi, Taiwan	1999	7.6	4.9	0.53	0.65	52.3	67.6
5	Chi-Chi, Taiwan	1999	7.6	11.5	1.16	0.42	114.8	45.6
6	Northridge, USA	1994	6.7	12.1	0.34	0.46	31.5	60.0
7	Northridge, USA	1994	6.7	5.3	0.60	0.84	77.5	130.4
8	Northridge, USA	1994	6.7	6.5	0.87	0.47	148.1	74.9
9	Northridge, USA	1994	6.7	7.0	1.58	1.29	53.9	103.3
10	Northridge, USA	1994	6.7	12.4	0.40	0.47	44.4	41.1
11	San Fernando, USA	1971	6.6	1.8	1.22	1.24	114.5	57.2
12	Tabas, Iran	1978	7.4	2.1	0.85	0.86	100.2	122.9
13	Loma Prieta, USA	1989	6.9	3.9	0.57	0.61	96.1	51.6
14	Cape Mendocino, USA	1992	7.0	8.2	0.59	0.66	48.4	88.7
15	Kobe, Japan	1995	6.9	1.0	0.83	0.63	91.1	76.1
16	Kobe, Japan	1995	6.9	1.5	0.62	0.67	120.8	123.2
17	Landers, USA	1992	7.3	2.2	0.73	0.79	133.7	28.2
18	Imperial Valley, USA	1979	6.5	1.4	0.45	0.45	67.1	113.6
19	Imperial Valley, USA	1979	6.5	0.6	0.34	0.47	51.6	113.2
20	N. Palm Springs, USA	1986	6.1	4.0	0.69	0.67	66.1	28.0
Note: *R*_rup_ is the closest distance to fault rupture								

**Fig. 9:**
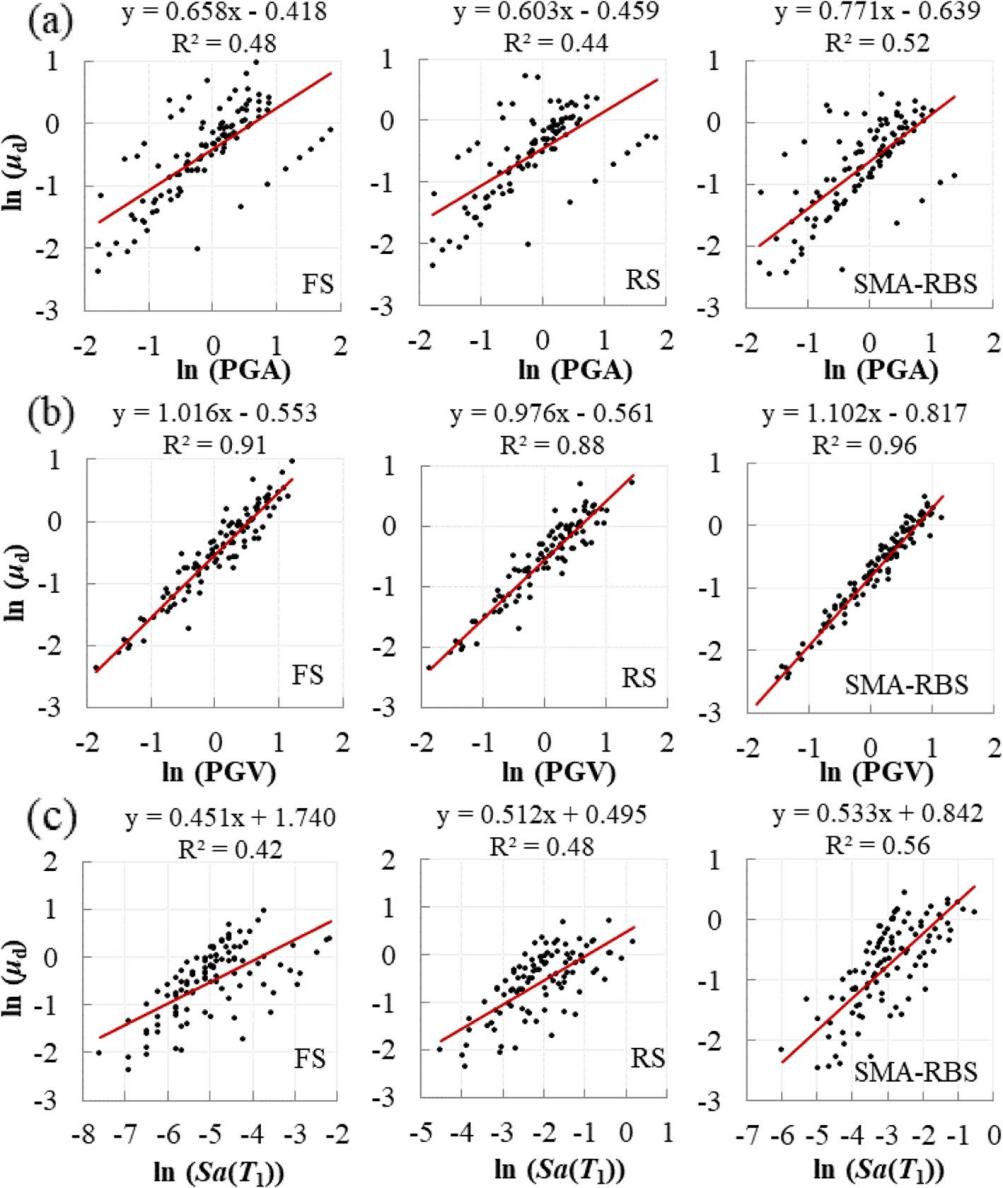
Probabilistic seismic demand models (PSDM) for displacement ductility of pier considering different IM candidates (**a**) PGA, (**b**) PGV, and (**c**) *Sa*(*T*_1_)

Mackie and Stojadinović (2007) suggested that the linear consistency between IM and EDP could be an indicator to select the optimal IM. According to this criterion, three widely used IMs, i.e. PGA, PGV, and *Sa*(*T*_1_) are examined to determine the optimal IM in this study. For simplicity, in Fig. 9, the displacement ductility of the bridge pier is plotted versus PGA, PGV, and *Sa*(*T*_1_) in the logarithmic form for four different bridge systems. The *R*^2^ values of the regression lines between EDPs and IM candidates are listed in Table 3. It can be observed that when PGV is selected as IM, *R*^2^ values are larger than 0.70, which reveals that the relationship between *ln*(PGV) and *ln*(EDP) is almost linear. Therefore, the PGV is considered as the IM.

**Table 3:** *R*^2^ in PSDMs for bridge components

EDP	*R*^2^ (FS)			*R*^2^ (RS)			*R*^2^ (SMA-RBS)		
	PGA	PGV	*Sa*(*T*_1_)	PGA	PGV	*Sa*(*T*_1_)	PGA	PGV	*Sa*(*T*_1_)
*ε*/*ε*_y_	0.56	0.76	0.25	0.39	0.77	0.55	0.57	0.79	0.33
*μ*_d_	0.48	0.91	0.42	0.44	0.88	0.48	0.52	0.96	0.56
*θ*	0.70	0.88	0.29	0.52	0.94	0.57	0.70	0.88	0.33
Δ	0.13	0.76	0.65	0.35	0.76	0.61	0.30	0.81	0.69

## Results and discussions

Since bi-directional shaking is considered for the bridge in this study, the responses of each bridge component should be calculated by considering the responses in both longitudinal (*x*) and transverse (*y*) directions.

9 $$R=\sqrt{{R}_{L}^{2}+{R}_{T}^{2}}$$

where, *R*_L_ and *R*_T_ are responses of the bridge, e.g. displacement of the pier, and drift ratio of the tower, in longitudinal and transverse directions, respectively.

For the bearings, the higher value of peak displacement in *x* and *y* directions, Δ_p_, is chosen as the indication of the capacity of bearings (Hedayati Dezfuli and Alam 2016). Hence, Δ_p_ may occur in either *x* or *y* direction based on the earthquake components.

10 $${\Delta }_{p}=\mathrm{max }\left({\Delta }_{{x}_{\mathrm{max}}},{\Delta }_{{y}_{\mathrm{max}}}\right)$$

## PSDM

The fragility functions of four bridge components, i.e. cable, pier, tower, and bearing, are developed in the incremental dynamic (IDA) analyses. For all bridge systems, *ln*(*a*), *b*, and *β*_D|IM_ are presented in Table 4.

**Table 4:** Regression coefficients of PSDMs for bridge components

Bridge System	Bridge Component	ln(*a*)	*b*	*β*_D/PGV_
FS	Cable	-0.946	0.047	0.017
	Pier	-0.553	1.016	0.221
	Tower	-5.160	0.909	0.232
	Roller	4.369	0.964	0.362
RS	Cable	-0.953	0.040	0.014
	Pier	-0.561	0.976	0.235
	Tower	-4.777	0.955	0.162
	Roller	3.778	0.966	0.646
SMA-RBS	Cable	-0.935	0.051	0.018
	Pier	-0.817	1.102	0.147
	Tower	-5.166	0.924	0.225
	SMA-RB	3.738	0.869	0.708

### Fragility of the components

#### Bearing

**Fig. 10:**
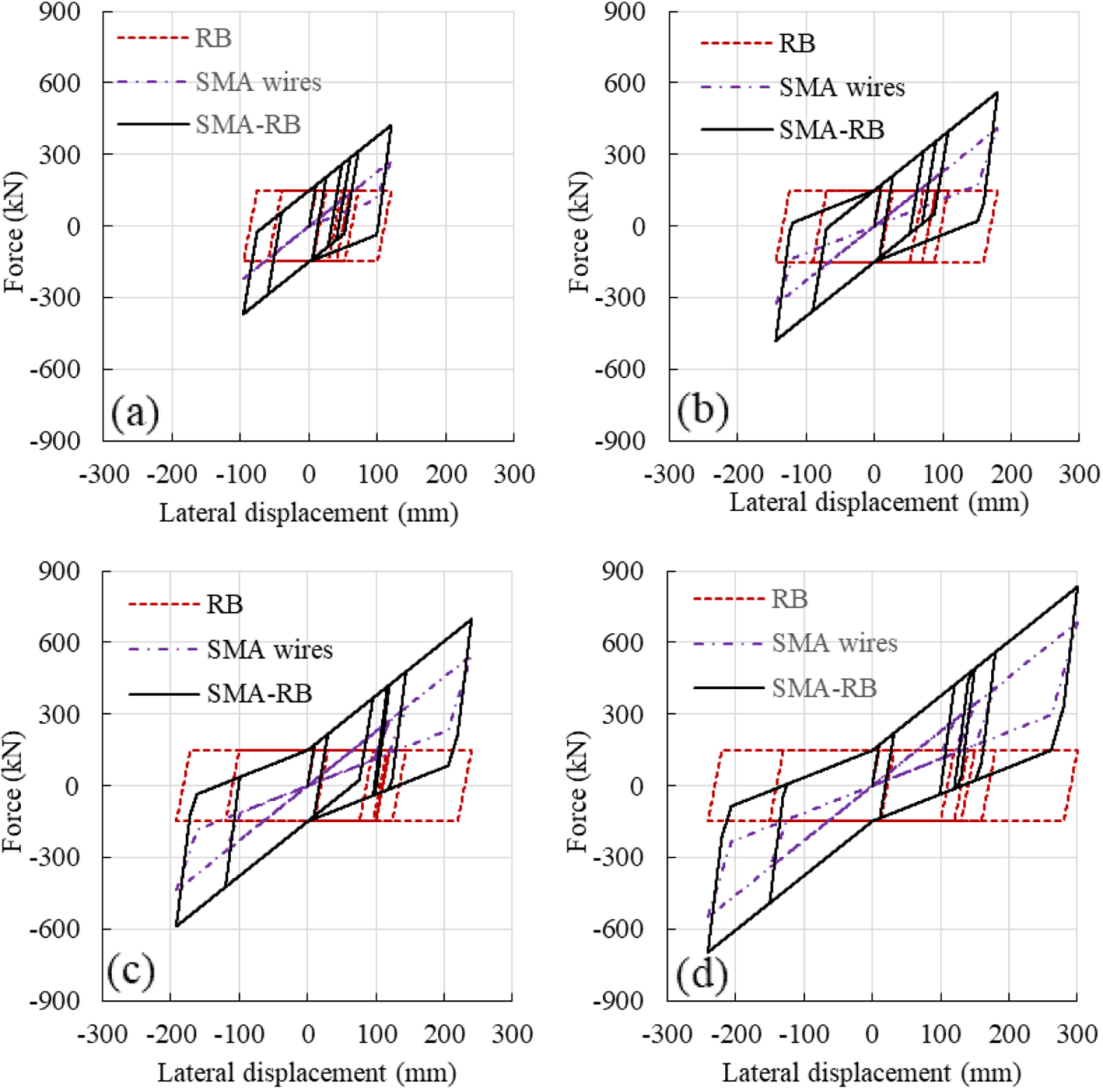
Typical force displacement responses of SMA-RBs at different damage states (**a**) Slight; (**b**) Moderate; (**c**) Severe; (**d**) Collapse

In order to clarify the work state of the SMA wires under earthquake, the typical force–displacement responses of the SMA-RB at different damage states are illustrated in Fig. 10. It can be calculated that the axial strains of SMA wires at slight, moderate, severe, and collapse damage states are 1.74%, 3.89%, 6.85%, and 10.65%, respectively, which is smaller than the superelastic strain of FeNiCoAlTaB SMA wires. The SMA wires can be functional before the collapse of the roller bearing.

**Fig. 11:**
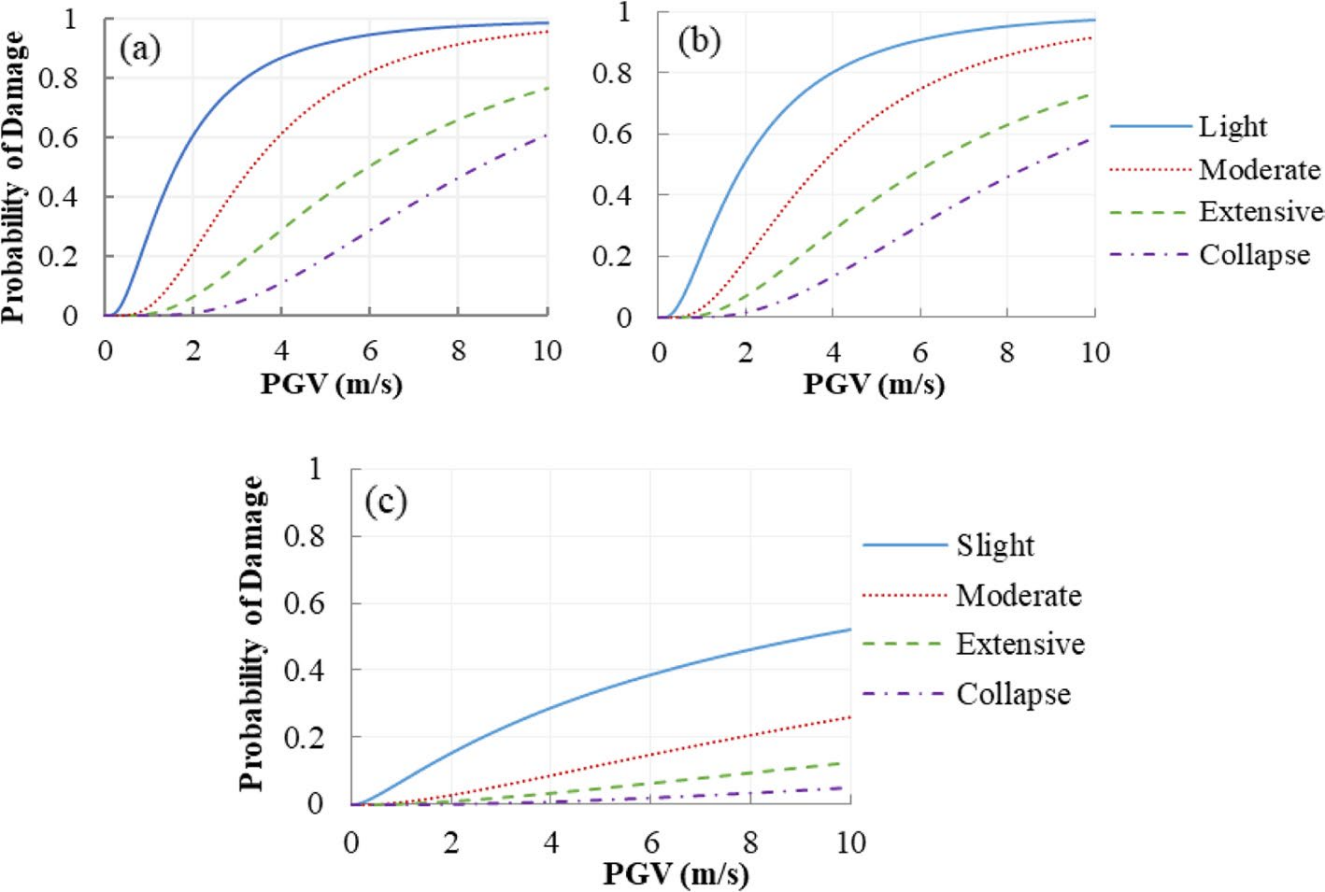
Fragility curves of the bearings (**a**) Floating System, (**b**) Rigid System, and (**c**) SMA-RBS

The fragility curves of the bearings at four damage states are shown in Fig. 11. From the design point of view (i.e. considering the worst-case scenario), the most vulnerable bearing is chosen to calculate the fragility functions. Since the counterweights in two side spans (i.e. bridge pier locations) are used to balance the weight of the main span, the inertia force transmitted to the bearing at the bridge pier location is larger than that at the tower location. Hence, the bearing at the bridge pier location is selected as the most vulnerable bearing.

It can be observed that the SMA wires in roller bearing can significantly reduce the vulnerability of the roller bearing at four damage states due to the increase of the lateral stiffness of the roller bearing. As an example, at the moderate limit state, the damage probability of SMA-RB is reduced by 85.9% compared to the roller supports in FS, at 4 m/s PGV. The possibility of failure decrease by 92.0% in the collapse state. The reason is that the longitudinal stiffness of the roller supports is much smaller than that of SMA-RBs. Another point is that the damage possibility of the bearings in RS is a little smaller than that of the bearings in FS. It can be understood that the hinge supports at the deck-tower connection in RS increase the bridge lateral stiffness and consequently, restrict the deck displacement at the pier locations.

#### Bridge tower

**Fig. 12:**
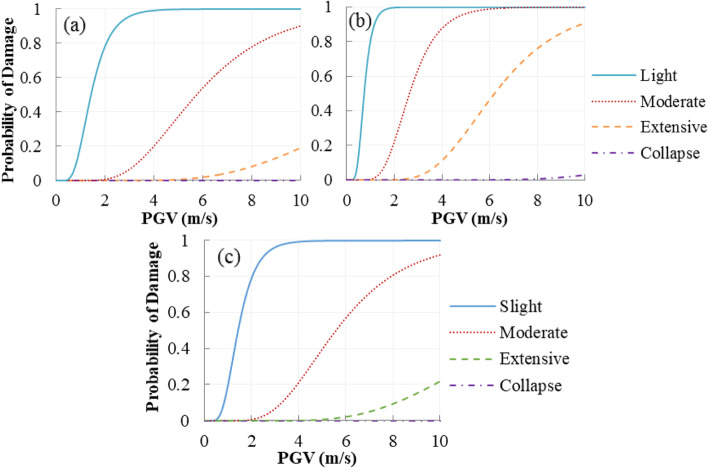
Fragility curves of bridge tower (**a**) Floating System, (**b**) Rigid System, and (**c**) SMA-RBS

Figure 12 illustrates the fragility curves of the bridge tower (left tower). It can be observed that the tower experiences a high probability at the slight damage state, whereas it has a low probability of occurring collapse damages under near-fault ground motions. When hinge supports are used to connect the bridge deck with the tower, i.e. the rigid system (RS), the damage probabilities of the tower are much higher than three other bridge systems. When the roller bearings in FS are used to replace the hinge supports, the damage probabilities of the bridge tower decrease at each limit state. It can be attributed to the fact that using the isolation systems can reduce the lateral stiffness of the deck-tower connection, compared to hinge supports, and as a result, a lower seismic force produced by the bridge deck is transmitted to the tower. When the SMA-RB is used to replace the roller and hinge bearings, compared to the tower in FS, the failure probabilities of the tower slightly increase at each limit state. This can be explained by the following fact. When SMA wires are implemented in the roller bearing, the bearing stiffness increases, and consequently, the seismic force demand has an increase.

As shown in Fig. 12, increasing the lateral stiffness of the deck-tower connection induced by the isolation bearings does not cause a sudden increase in the vulnerability of the tower, compared to FS. Both stiffness and energy dissipation capacity of the isolation systems contributes to the fragility of the tower. Increasing the stiffness of the deck-tower connection leads to an increase in the vulnerability of the tower, whereas its fragility will decrease with the increase of the energy dissipation of the isolation bearings. Here, the stiffness of the isolators is much smaller than that of the tower with a huge cross-section. Hence, when the SMA-RBs are used, the influence of the isolators’ stiffness is less than or close to that of the energy dissipation capacity. For instance, at the slight damage state, for a PGV of 4 m/s, the probabilities of damage are 99.3% in the tower equipped with SMA-RBs, and 100% and 99.1% for the tower in RS and FS, respectively. For a PGV of 6 m/s, the towers in SMA-RBS and FS experience 57% and 54% damage probability at the moderate damage level, whereas the damage probability of the tower in RS is 98.9% at the same damage level.

#### Bridge pier

**Fig. 13:**
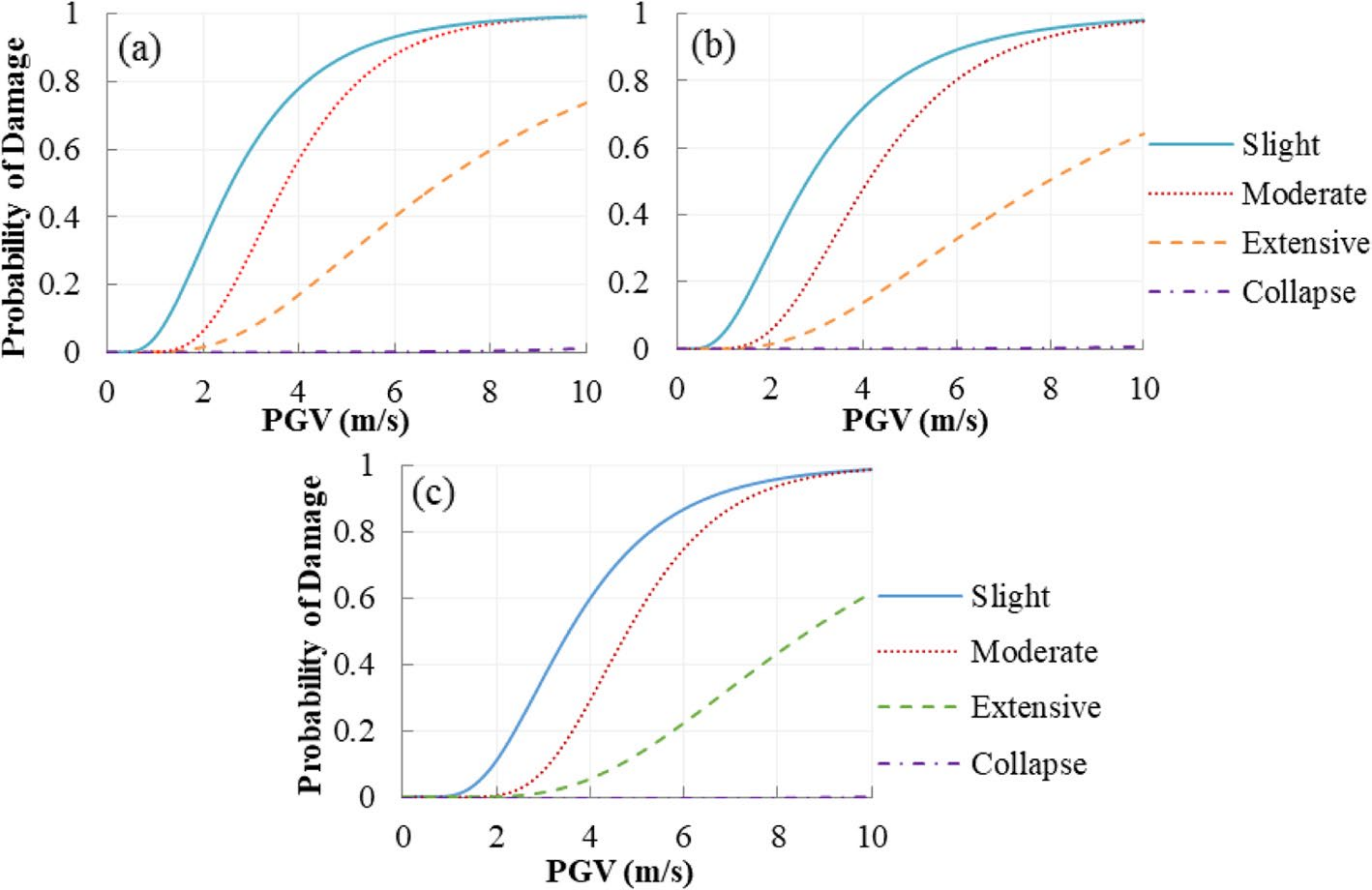
Fragility curves of bridge pier (**a**) Floating System, (**b**) Rigid System, and (**c**) SMA-RBS

Figure 13 shows the fragility curves of the most vulnerable bridge pier, i.e. the transition pier. The bridge pier in FS experiences higher probabilities of damage compared to those in RS. The bridge piers equipped with SMA-RBs have lower damage probabilities, compared to the piers equipped with roller bearings. For example, the possibilities of reaching slight, moderate, and extensive damages in the pier equipped with roller bearings in FS at a PGV of 6 m/s are 93.2%, 87.9%, and 40%, respectively, while the corresponding probabilities of using SMA-RBs are 86.7%, 74.7%, and 22.4%, respectively. It can be explained that since SMA-RB can dissipate more seismic energy, it will noticeably decrease the damage probability of the bridge pier, compared to the roller bearings.

#### Bridge cable

**Fig. 14:**
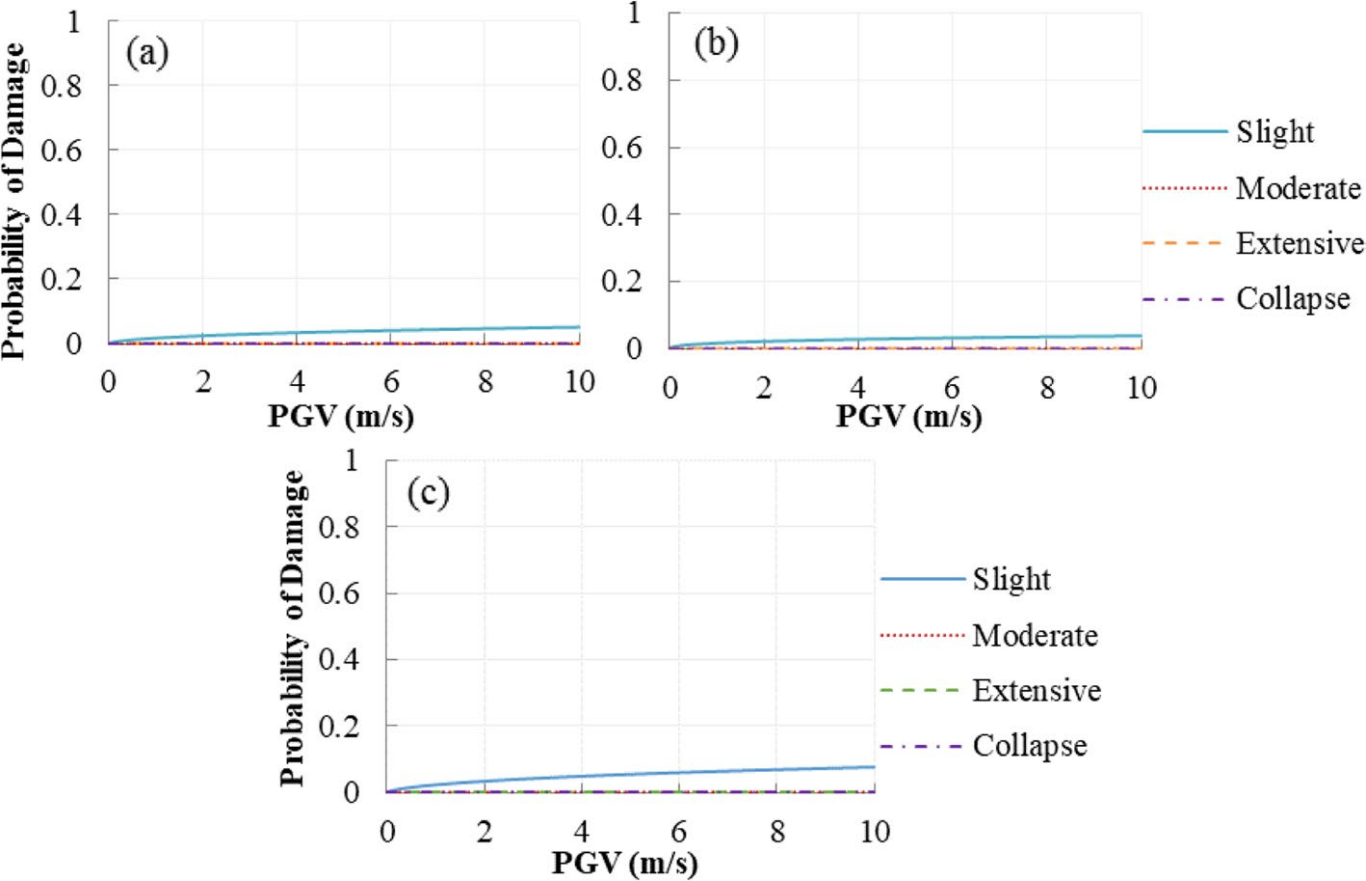
Fragility curves of cable (**a**) Floating System, (**b**) Rigid System, and (**c**) SMA-RBS

Since the longest cable experiences the highest responses compared to the other cables, cable 1 is selected as the most vulnerable cable. The fragility curves of the cable are illustrated in Fig. 14. The cable is the least vulnerable component compared to the towers, piers, and bearings. Only failure probabilities of the cable at the slight damage state can be observed. It means that the cables perform well and remain elastic under near-fault ground motions. Since smart isolation bearings can increase the vertical stiffness of the deck-tower and deck-pier connections, SMA-RB makes the cable more vulnerable at the slight damage state. As an example, the probabilities of the cable in FS, RS, and SMA-RBS at a PGV of 6 m/s are 4.1%, 3.2%, and 5.8%, respectively.

Another important finding is that the bridge pier, tower, and cable in the four bridge systems have a very low failure probability (smaller than 1.5% at a PGV of 10 m/s) at the collapse damage level. It is consistent with the fact that as a lifeline facility, the cable-stayed bridge is designed to withstand any earthquake without collapse.

### System fragility

**Fig. 15:**
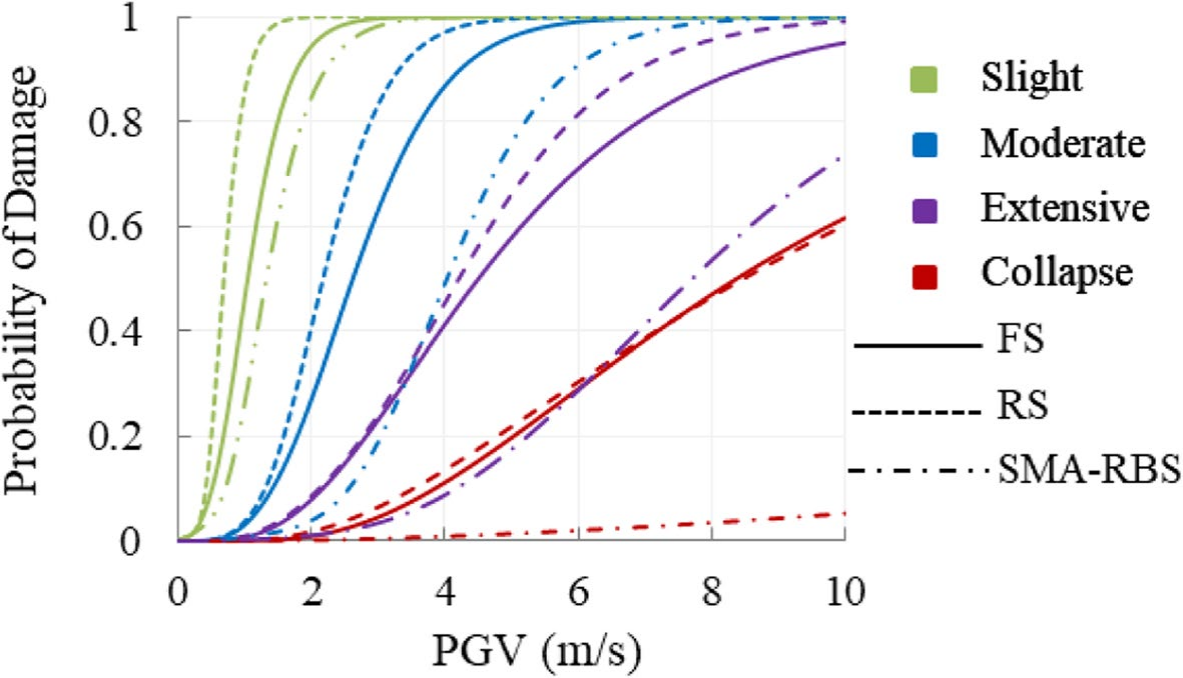
Fragility curves of the bridge system with or without isolation system

The maximum envelope of the upper bounds of the serial and parallel system is used to derive the system fragility. To compare the performances of the reference bridges and the bridge equipped with SMA-RBs, three sets of fragility curves are illustrated in Fig. 15. Results show that when hinge supports are used as the deck-tower connections, i.e. the rigid system (RS), the bridge has the highest damage probability compared to the other bridge systems. The floating system (FS) is the second most vulnerable system. After installing the smart isolation system, the bridges with SMA-RBs have lower damage possibilities.

Failure probabilities of FS, RS, and SMA-RBS are presented in Table 5 for three values of PGV (2, 6, and 10 m/s). Since RS is the most vulnerable bridge system, the damage likelihood of FS and SMA-RBS is compared with that of RS. At a PGV of 6.0 m/s, the probabilities of FS at moderate, extensive, and collapse damages, respectively are 0.9%, 12.8%, and 4.6% lower than that of RS. When SMA-RBs are used to replace the hinge supports, the corresponding values of SMA-LRBS increase to 9.1%, 64.6%, and 93.4%, respectively. At collapse level and a PGV of 10.0 m/s, the fragility of the bridges equipped with SMA-LRBs is 91.2% lower than that of RS. At a PGV of 6.0 and 10.0 m/s, the damage probabilities of the SMA-LRBS undergoing extensive damage are 28.8% and 73.7%, respectively. Under the same situation, the damage probabilities of the SMA-RBS undergoing collapse damage decrease to 2% and 5.3%, respectively. This result indicates that SMA-RB is more efficient when the bridge experiences large displacement at collapse damage states.

**Table 5:** Damage probabilities of the bridge system with or without isolation system

PGV (m/s)	Bridge system	Slight		Moderate		Extensive		Collapse	
		P	∆	P	∆	P	∆	P	∆
2	RS	0.999	─	0.407	─	0.086	─	0.018	─
	FS	0.943	-5.6%	0.270	-33.7%	0.079	-8.1%	0.009	-50.0%
	SMA-RBS	0.845	-15.4%	0.039	-90.4%	0.011	-87.2%	0.002	-88.9%
6	RS	1.000	─	0.999	─	0.814	─	0.304	─
	FS	1.000	0.0%	0.990	-0.9%	0.710	-12.8%	0.290	-4.6%
	SMA-RBS	1.000	0.0%	0.908	-9.1%	0.288	-64.6%	0.020	-93.4%
10	RS	1.000	─	1.000	─	0.991	─	0.602	─
	FS	1.000	0.0%	1.000	0.0%	0.950	-4.1%	0.616	2.3%
	SMA-RBS	1.000	0.0%	0.999	-0.1%	0.737	-25.6%	0.053	-91.2%

Note: ∆ is the relative difference between the rigid system (RS) and two other bridge systems

## Conclusions

This paper numerically assesses the seismic fragility of a cable-stayed bridge equipped with new type of seismic isolator, i.e. SMA-RBs. The hysteretic model of SMA-RB is analytically developed and implemented in OpenSees, which has been validated against the experimental results. The seismic fragilities of three bridge systems are evaluated at component and system level under 20 near-fault ground motions. The main conclusions are summarized as follows:

- (1) A framework is introduced to develop the system fragility of cable-stayed bridges equipped with SMA-RBs. Simultaneously, a procedure is proposed to predict the damage states for SMA-RB, which is useful for the designers to design the smart bearings based on the desired performance level.
- (2) The applicability of three IMs, i.e. PGA, PGV, and *Sa*(*T*_1_), are compared and the PGV is selected as the optimal IM for the long-span cable-stayed bridge because a better (almost linear) relation can be achieved between *ln*(PGV) and *ln*(EDP).
- (3) The piers and towers equipped with SMA-RBs have lower damage probabilities compared to the piers and towers equipped with conventional bearings. SMA-RBs are less fragile than the convention bearings (i.e. roller supports). SMA-RBs may make the cable more vulnerable at the slight damage state.
- (4) The bridge pier, tower, and cable in the four bridge systems have a low damage probability (smaller than 1.5% at a PGV of 10 m/s) at the collapse level. It means that the considered cable-stayed bridge can withstand severe earthquakes without collapse.
- (5) SMA-RBs can significantly decrease the overall fragility of the whole bridge system compared to roller bearings. It is found that SMA-RB is more efficient when the bridge system experiences large amplitude vibration. The SMA-RBS experiences a much lower collapse damage probability compared to RS ad FS.

The geometrical and material uncertainties and the spatial variability of ground motions were not considered in the fragility study when assessing the seismic performance of the bridge equipped with SMA-RBs. To comprehensively assess the vulnerability of such novel bridges, these factors should be considered in future works.

## Data Availability

The data and materials in the current study are available from the corresponding author on a reasonable request.
